# Examining the Influenza A Virus Sialic Acid Binding Preference Predictions of a Sequence‐Based Convolutional Neural Network

**DOI:** 10.1111/irv.70044

**Published:** 2024-12-11

**Authors:** Laura K. Borkenhagen, Jonathan A. Runstadler

**Affiliations:** ^1^ Department of Infectious Disease and Global Health, Cummings School of Veterinary Medicine Tufts University North Grafton Massachusetts USA

**Keywords:** hemagglutinin, influenza, machine learning, receptor binding

## Abstract

**Background:**

Though receptor binding specificity is well established as a contributor to host tropism and spillover potential of influenza A viruses, determining receptor binding preference of a specific virus still requires expensive and time‐consuming laboratory analyses. In this study, we pilot a machine learning approach for prediction of binding preference.

**Methods:**

We trained a convolutional neural network to predict the α2,6‐linked sialic acid preference of influenza A viruses given the hemagglutinin amino acid sequence. The model was evaluated with an independent test dataset to assess the standard performance metrics, the impact of missing data in the test sequences, and the prediction performance on novel subtypes. Further, features found to be important to the generation of predictions were tested via targeted mutagenesis of H9 and H16 proteins expressed on pseudoviruses.

**Results:**

The final model developed in this study produced predictions on a test dataset correctly 94% of the time and an area under the receiver operating characteristic curve of 0.93. The model tolerated about 10% missing test data without compromising accurate prediction performance. Predictions on novel subtypes revealed that the model can extrapolate feature relationships between subtypes when generating binding predictions. Finally, evaluation of the features important for model predictions helped identify positions that alter the sialic acid conformation preference of hemagglutinin proteins in practice.

**Conclusions:**

Ultimately, our results provide support to this in silico approach to hemagglutinin receptor binding preference prediction. This work emphasizes the need for ongoing research efforts to produce tools that may aid future pandemic risk assessment.

## 1 Introduction

Affinity for specific conformations of the terminal sialic acid is regarded as a determinant of host tropism for influenza A viruses (IAVs); IAVs from human hosts tend to have a higher affinity to α2,6‐linked sialic acids, while IAVs from avian hosts tend to have a higher affinity to α2,3‐linked sialic acids [1]. While there are many amino acid changes to the receptor binding protein hemagglutinin (HA) that have been found to result in a change in affinity from α2,3‐linked to α2,6‐linked sialic acid receptors [2], the single and combinatorial amino acid changes that lead to this switch are not comprehensively known, particularly for subtypes that historically have not caused outbreaks of infections in humans.

In this study, we developed a convolutional neural network (CNN) that predicts the receptor binding specificity of IAV given the HA amino acid sequence using binding data generated in house and collected from the Consortium for Functional Glycomics (CFG) [3]. Such a tool may be useful for identifying IAVs collected during surveillance efforts that may warrant further study for evidence of viral transmission between avian and mammalian hosts that present a risk of spillover to humans. To assess whether the model generates predictions based on meaningful amino acid sequences features, the importance of amino acids for generating predictions was derived and used to inform targeted mutagenesis in search of binding class changes. The limits of this CNN modeling approach were also tested through an evaluation of performance over test sequences with varying degrees of missing amino acids. Finally, a CNN was trained using the same approach without H16 sequences in the training data to evaluate the predictive performance of this modeling approach on a novel sialic acid‐binding HA type. In all, this study provides a model that accurately predicts the sialic acid conformation binding preference of IAVs and helps to identify areas for future research based on the limitations observed during model testing.

## 2 Methods

The training and testing data used in the modeling comes from two sources: viruses and pseudoviruses collected and tested in the Runstadler laboratory and data collected from the CFG. The phylogenetic relationships of the 358 training samples and 48 test samples are visualized in Figure S1, and sample size details are available in Table 1.

**TABLE 1:** Summary of training data and independent test data.

Sample type				
	Active virus	Inactive virus	Isolated protein	Pseudovirus
Train	186	1	134	37
Test	38	0	0	10

Host class			
	Avian	Mammalian	Other/unknown
Train	143	134	81
Test	40	8	0

Virus subtype																
	H1	H2	H3	H4	H5	H6	H7	H8	H9	H10	H11	H12	H13	H14	H15	H16
Train	72	15	66	14	54	14	31	7	14	15	13	9	16	6	2	10
Test	3	3	3	3	3	3	3	3	3	3	3	3	3	3	3	3

Receptor specificity		
	No preference for α2,6‐linked sialic acid	Preference for α2,6‐linked sialic acid
Train	277	81
Test	39	9

### 2.1 Modeling Data Generated in House

#### 2.1.1 Viruses and Plasmids

In total, 197 IAV isolates, RNA, or HA plasmids were obtained for use in this study (sources in Table S1). The sequences of all HA genes from viruses or plasmids were confirmed via nanopore sequencing (Plasmidsaurus, Eugene, OR, USA). Of these, 149 were used for model training and 48 were used as an independent test dataset.

#### 2.1.2 Pseudovirus Generation

To generate receptor binding results for IAVs where only RNA or HA plasmids were available, a lentiviral vector approach for pseudovirus generation was employed. Human embryonic kidney (HEK)‐293T cells cultured in DMEM high glucose with GlutaMAX (Thermo Scientific, Waltham, MA, USA) supplemented with 10% heat inactivated fetal bovine serum, 1% penicillin–streptomycin, and up to 2.5 μg/mL amphotericin B. At 60% confluence, HEK293T cells were transfected with plasmids expressing HIV Gag with GFP (National Institutes of Health, HIV Reagent Program, Manassas, VA, USA) and HA using Lipofectamine 3000 (Invitrogen, Carlsbad, CA, USA) per manufacturer instructions. At 24 and 48 h post transfection, spent culture media was removed, and *Vibrio cholerae* neuraminidase (Type III, Sigma, St. Louis, MO, USA) supplemented culture media (7 mU/mL) was added to free the newly budded pseudoviruses from the host cells. At 48 and 72 h, the media was harvested, centrifuged, and filtered to remove cellular debris and concentrated by ultracentrifugation at 77,000 *g* for 2 h at 4°C with a 1‐mL 20% sucrose in phosphate buffered saline (PBS) cushion. Concentrated pseudoviruses were resuspended overnight in 250‐μL PBS.

#### 2.1.3 Modified Hemagglutination Assay

A modified hemagglutination assay was used to determine the receptor binding specificity of the IAV isolates and pseudoviruses [4]. Briefly, 62.5 μL of 20% turkey red blood cells (RBCs; Lampire Biological Laboratories, Pipersville, PA, USA) were desialylated with 12.5 mU of *Vibrio cholerae* neuraminidase (Type III) for 1 h at 37°C. The desialylated RBCs were selectively resialylated with 4.4 μg of α2,3‐sialyltransferase (ST3Gal6; R&D Systems, Minneapolis, MN, USA) or 2.7 μg of α2,6‐sialyltransferase (ST6Gal1; R&D Systems, Minneapolis, MN, USA) and 1.5‐mM CMP‐sialic acids (Sigma, St. Louis, MO, USA) with 1% bovine serum albumin (BSA) in PBS in a total volume of 75 μL for 2 h at 37°C. The untreated, desialylated, and sialyltransferase‐treated RBCs were washed with PBS and brought to a final concentration of 0.5% in PBS with 1% BSA. The viruses and pseudoviruses were serially diluted in PBS (two‐fold) and mixed in equal parts with 0.5% treated or untreated RBCs and allowed to incubate at room temperature for 30 min. All samples were tested in duplicate.

### 2.2 Modeling Data Collected From CFG

#### 2.2.1 Data Collection

Glycan binding profiles from any array version (1 through 5.2, containing 200–609 glycans, respectively) of IAVs or purified HA proteins were collected from CFG in May 2022 [3]. The corresponding HA amino acid sequence for each binding profile was provided by the submitting investigator or collected from the Influenza Research Database (IRD) [5] by the accession number or strain name. Entries completely missing binding or sequence data were removed. Binding profiles with the most extensive binding data were selected in cases of sequences with multiple submissions. This resulted in a total of 209 binding profiles with complete HA sequences, which were used for downstream analyses. Additionally, 42,416 and 50,241 unique and complete HA amino acid sequences with associated metadata were obtained from the IRD [5] and the Global initiative on Sharing All Influenza Data (GISAID) [6], respectively, for use in model training.

#### 2.2.2 Defining Binding Labels

For each binding profile, the dominant glycans bound by each virus or HA protein were determined using a method from Grant et al. [7] whereby the glycans having a signal at least 10% of the maximum affinity signal for that virus or protein were considered dominant binders. Comparisons of binding affinity were made by pairing the sialylated glycans present in the binding arrays such that each pair differed only the in terminal sialic acid conformation (glycan pairings are available in Table S2). Welch's *t*‐test was used to identify a significant difference (*p* < 0.05) in binding affinity between each glycan pair. Sequences were labelled as showing a preference for α2,6‐linked sialic acid receptors or not if there was a significant difference in affinity within all binding pairs meeting the dominant binder threshold.

### 2.3 Modeling

#### 2.3.1 Data Encoding

A heterosubtypic alignment of all sequences used in this study was performed per the method published by Burke and Smith [8] using MAFFT [9] and Jalview [10]. The aligned sequences were coded with a binary indication of the presence or absence of each amino acid at each position in the sequence (one‐hot encoding) using Sklearn [11].

#### 2.3.2 CNN Architecture

The model used in this study was adapted from a tailored CNN developed by Scarafoni and colleagues to identify avian versus human‐origin influenza virus sequences [12] and performed using Keras [13]. The CNN was comprised of five convolutional layers, where each layer detects specific patterns in the input data or features extracted from the preceding layer. These patterns are identified by passing filters over the data, the number and size of which determines the range and complexity of the patterns recognized. For each convolutional layer, a ReLU function is applied to enhance the ability of the network to capture relationships within the data. Following each convolutional layer is a maxpooling layer which reduces the spatial dimensions of the data by selecting the maximum value within each “window” (or kernel) of data. The size of the kernel and the amount by which it moves along the data (stride) determines how much the data dimensionality is reduced. Batch normalization was performed after each maxpooling layer to standardize and rescale the data. After these layers, the data is flattened and passed through two fully connected layers where the model associates the features extracted from the earlier layers with the target host class. A sigmoid activation function was applied in the final layer to produce a probability distribution ranging from 0 to 1 for classification of the samples.

**FIGURE 1:**
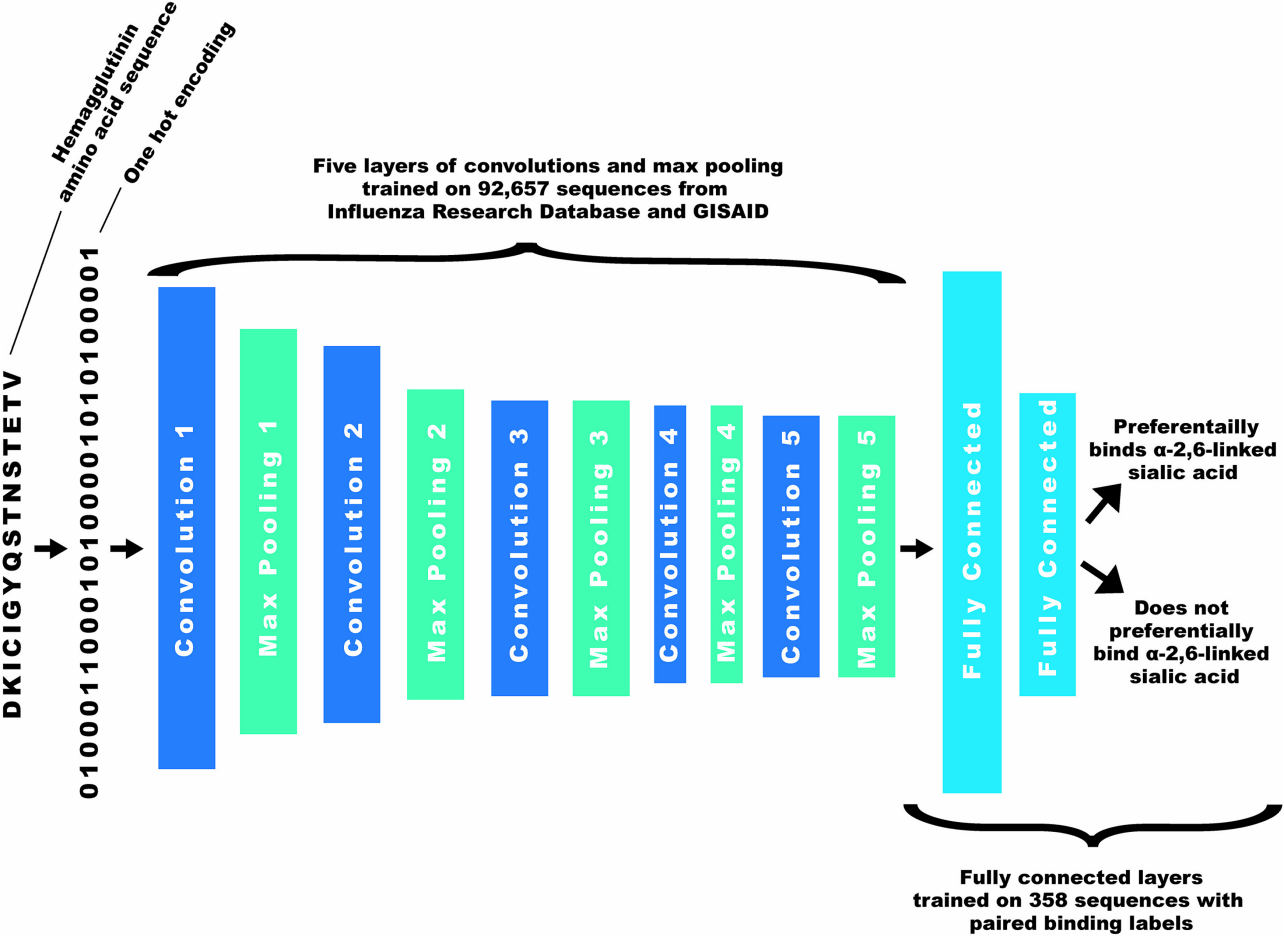
Simplified convolutional neural network architecture.

The host prediction model was trained with the sequences from IRD [5] and GISAID [6] to recognize those of avian or mammalian origin. Model training was performed by passing batches of 128 training samples through at a time, calculating the binary cross‐entropy loss function which quantifies the difference between the predicted probability and actual label of the data, and updating, through backpropagation, the model parameters via Adam optimization. During training, a dropout rate of 0.5 was applied to prevent overfitting by randomly dropping out half of the connections between nodes before the final fully connected layer in each training iteration. The samples were class and subtype weight biased to account for differences in training data representation. Training was stopped using an early stopping strategy, which tracks the model performance on a 20% held out validation set of samples to identify when prediction performance peaks during model training, thereby preventing overfitting of the training data. The trained origin host model showed an AUC of 0.99 and accuracy of 0.99 on the validation set. The weights of the convolutional and maxpooling layers of the origin host CNN were frozen, and new fully connected layers were trained with binding data from 358 samples with subtype and class weighting. Early stopping with a 5% held out validation set was used to prevent overtraining. A simplified illustration of the final model is shown in Figure 1 and details of the model parameters and hyperparameters are available in Table S3. Hyperparameters tested included the Adam learning rate (0.1, 0.01, 0.001, 0.0001) and batch size (8, 16, 32, 64) which led to optimum performance at 0.01 and 32, respectively.

#### 2.3.3 Testing Model Performance

The AUC and accuracy of the model was determined by generating predictions on a test data set of 48 samples, 3 of each subtype, covering all major sialic acid binding subclades (Figure S1). To assess the impact of missing data on test predictions, a random stretch of amino acids was coded as missing data (10, 20, 40, 80, 160, and 320 amino acids) in each test sequence. Predictions were generated over 100 bootstraps per amount of missing data. The mean and standard deviation of the test AUC and accuracy were recorded. Additionally, to evaluate the positions at which missing amino acids impact performance, a length of 80 ambiguous amino acids was coded into each possible start position of the test data prior to model prediction; AUC and accuracy were recorded. Finally, to assess the performance of this CNN modeling approach for novel HA‐type sialic acid receptor binding prediction, a new CNN was trained in the absence of H16 sequences in the base host model used for transfer learning and the labelled training data. Predictions were generated on the 48 test sequences and the sequences of H17, H18, and H19.

#### 2.3.4 Amino Acid Importance

Assessments of amino acid saliency in predicting binding labels were performed using Shapley additive explanations (SHAP values) on the independent test dataset [14]. These values are derived from cooperative game theory principles to assign a relative importance to each feature (presence or absence of an amino acid at each position) to the prediction the model generated (preference for α2,6‐linked sialic acids). In that way, it aids in the understanding of the contribution of each amino acid in the sequence to the decision‐making process of the model. The absolute means of the SHAP values across all test samples and amino acids for each position were calculated to assess global amino acid importance. SHAP values were also generated for specific H9 and H16 amino acid sequences to inform site directed mutagenesis targets.

### 2.4 Laboratory Follow‐Up of Model‐Determined Amino Acid Importance

#### 2.4.1 Amplification of Hemagglutinin

RNA was extracted from viral stocks of A/Guinea Fowl/Hong Kong/WF10/1999(H9N2) and A/shorebird/Delaware/172/2006(H16N3) with the Mag‐Bind Viral DNA/RNA extraction kit (Omega Biotek, Norcross, GA, USA) per manufacturer instructions. Reverse‐transcription of RNA into cDNA was performed using the qScript Ultra Flex Kit (Quantabio, Beverly, MA, USA) per manufacturer instructions with a final concentration of 1 μM of gene‐specific forward primers designed for assembly into a pcDNA3.1(+) (Invitrogen, Carlsbad, CA, USA) vector (Table S4). HA genes were amplified using the NEBNext Q5 Hot Start HiFi PCR Master Mix (New England BioLabs Inc., Ipswich, MA, USA) with a final concentration of 0.5 μM each of forward and reverse primers (Table S4) and 4 μL of cDNA template. Thermocycling conditions were as follows: initial denaturation at 98°C for 30 s, 35 cycles of denaturation at 98°C for 10 s followed by annealing/extension at 72°C for 90 s, and final extension at 72°C for 2 min. The amplified HA genes (~1.7 kilobases) were gel extracted with the QIAquick gel extraction kit (Qiagen, Hilden, Germany). The concentration of the gel extracted dsDNA was determined by Qubit (Invitrogen, Carlsbad, CA, USA), and the DNA sequences were confirmed by nanopore sequencing (Plasmidsaurus, Eugene, OR, USA).

#### 2.4.2 Molecular Cloning

The pcDNA3.1(+) vector was digested by NheI and BamHI restriction enzymes (New England BioLabs Inc., Ipswich, MA, USA) overnight, and the linearized vector was gel extracted. The linearized vector and amplified HA genes were mixed at a 1:2 DNA molar ratio (50‐ng vector + 31.7‐ng HA) with NEBuilder HiFi DNA Assembly Master Mix (New England BioLabs Inc., Ipswich, MA, USA) and incubated at 50°C for 15 min. The assembled plasmids were transformed into NEB Stable Competent *E. coli* (New England BioLabs Inc., Ipswich, MA, USA), plated on Luria‐Bertani (LB) agar with 100 μg/mL ampicillin, and incubated at 30°C overnight. Individual colonies were screened for the presence of the insert using OneTaq Hot Start Quick‐Load 2X Master Mix (New England BioLabs Inc., Ipswich, MA, USA) with primers outside of the insertion site (Table S4) at a final concentration of 0.2 μM using the following thermocycling conditions: initial denaturation at 94°C for 5 min, 30 cycles of denaturation at 94°C for 30 s followed by annealing at 52°C for 30 s and extension at 68°C for 2 min, and final extension at 68°C for 2 min. Colonies containing the appropriate‐sized insert were grown in a start culture of sterile LB broth with 100 μg/mL ampicillin. Flasks of 100–200 mL of sterile LB broth with 100 μg/mL ampicillin were inoculated with start culture at a 1:500 dilution. The final cultures were pelleted and maxipreps were performed using the E.Z.N.A. Endo‐Free Plasmid Maxi Kit (Omega Biotek, Norcross, GA, USA) per manufacturer instructions. The concentration of each plasmid was determined by Qubit, and the DNA sequences of the plasmids were confirmed to contain no changes to the HA insert by nanopore sequencing (Plasmidsaurus, Eugene, OR, USA).

#### 2.4.3 Site‐Directed Mutagenesis

Site‐directed mutagenesis was performed using the Q5 Site Directed Mutagenesis Kit (New England BioLabs Inc., Ipswich, MA, USA) per manufacturer instructions. Briefly, the plasmids bearing HA were amplified using primers with base changes (Table S5) per the following conditions: initial denaturation at 98°C for 30 s, 25 cycles of denaturation at 98°C for 10 s followed by annealing at a primer specific temperature (Table S5) and extension at 72°C for 3 min and 30 s, and final extension at 72°C for 2 min. The amplified PCR products were treated with Kinase‐Ligase‐DpnI and used to transform *E. coli* .

## 3 Results

### 3.1 Binding Predictions on Independent Test Dataset

**FIGURE 2:**
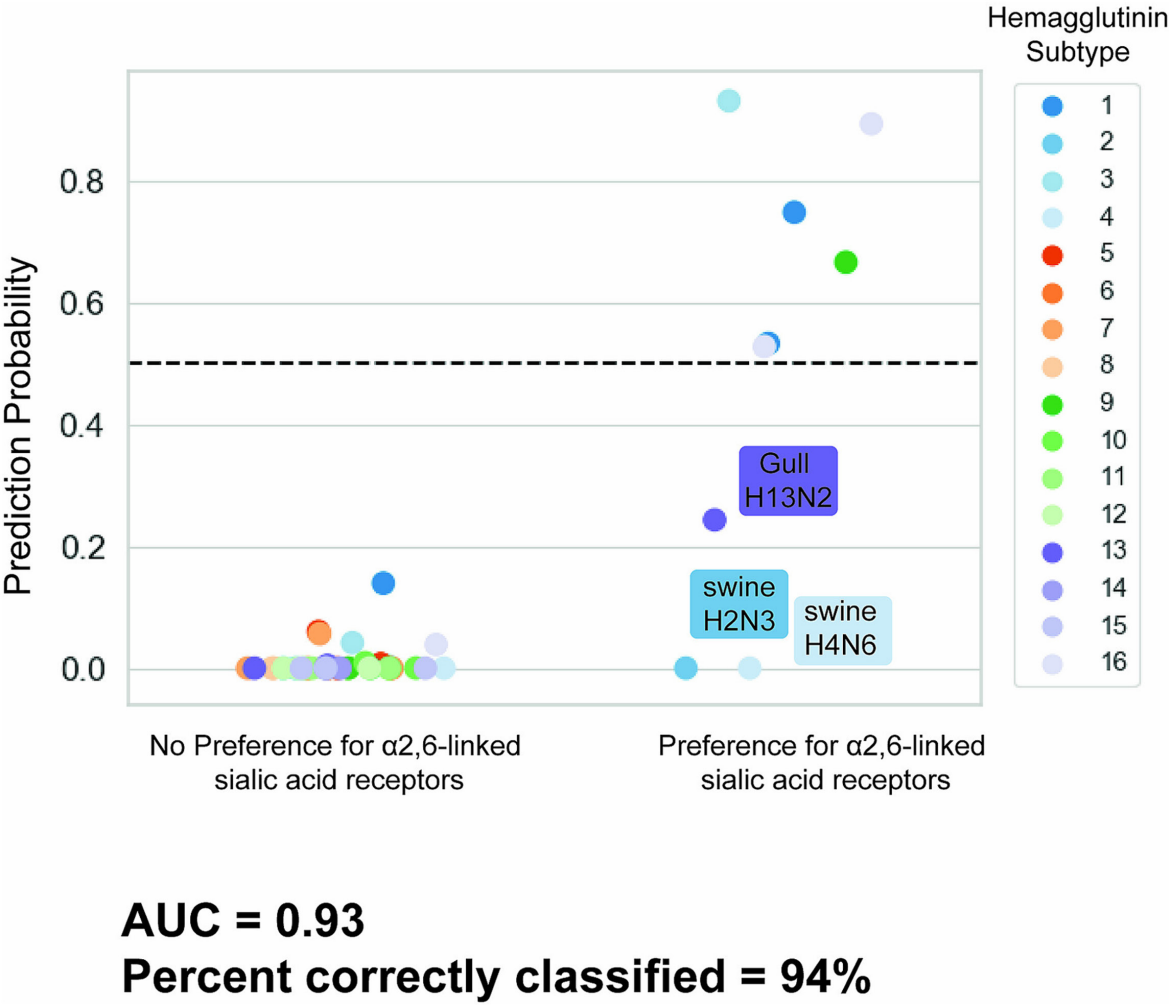
Probabilities predicted from an independent test of a convolutional neural network trained to classify hemagglutinin sequences by α2,6‐linked sialic acid binding preference.

On an independent test dataset, the model generated predictions correctly 94% of the time with an area under the receiver operating characteristic curve (AUC) of 0.93 (Figure 2). Samples that were correctly predicted not to preferentially bind α2,6‐linked sialic acid receptors were predicted with a greater certainty than those correctly predicted to prefer α2,6‐linked sialic acid receptors. Additionally, no samples that did not preferentially bind α2,6‐linked sialic acid receptors were misclassified (i.e., no false positives were predicted). Three of the test samples were incorrectly classified, including: A/swine/Missouri/4296424/2006(H2N3), A/swine/Missouri/A01727926/2015(H4N6), and A/herring gull/Massachusetts/A00080255/2006(H13N2). These are all false negative predictions. When broken down by HA group, group 1 HA (H1, H2, H5, H6, H8, H9, H11, H12, H13, and H16) binding was predicted with an AUC 0.96 and accuracy of 0.93, while group 2 HA (H3, H4, H7, H10, H14, and H15) binding was predicted with an AUC of 0.81 and accuracy of 0.94.

### 3.2 Amino Acids That Influence Binding Predictions

**FIGURE 3:**
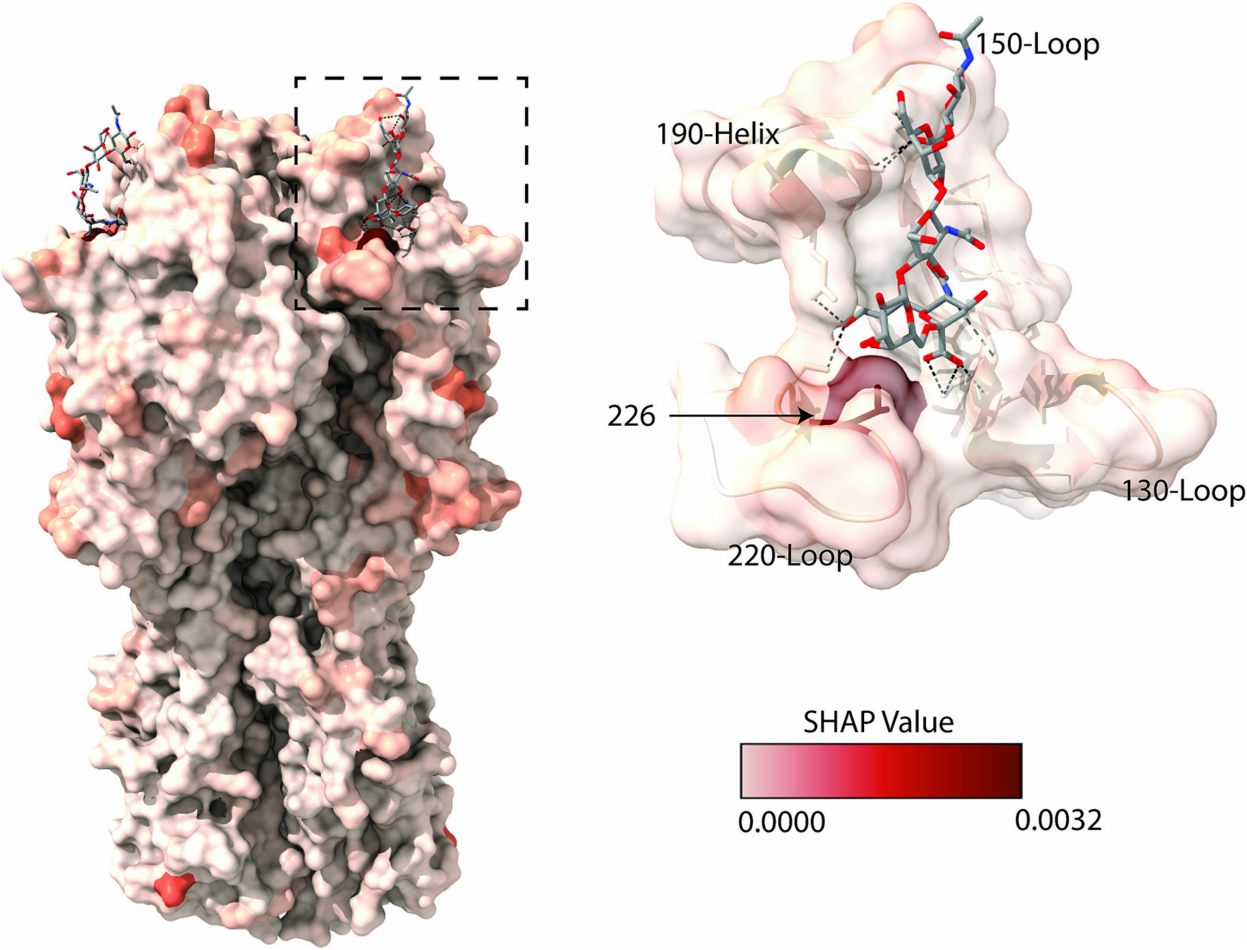
Saliency map of SHAP values rendered on a hemagglutinin protein. The values rendered are the absolute mean of SHAP values of a convolutional neural network trained to differentiate hemagglutinin sequences by binding preference to α2,6‐linked sialic acid receptors. The absolute means were taken for each amino acid position generated on an independent test dataset. The dotted line box indicates the region of a zoomed in view of the binding pocket (on the right). Important structures and residues are annotated in black. The depictions used PDB 6TZB and were generated in ChimeraX [15].

The highest magnitude absolute mean SHAP values for the test samples globally were more localized to the head of HA (HA1) over the stalk region (HΑ2) (Figure 3). The site with the highest absolute mean SHAP value was 226. To see whether binding switch predictions could be made with this model evaluation technique, viruses that violate the canonical tropism were selected for site directed mutagenesis, that is, avian‐derived IAVs that bind preferentially to α2,6‐linked sialic acid receptors. To understand which residues are important for conferring α2,6‐linked sialic acid preference, SHAP values for each amino acid at each position were generated for A/Guinea Fowl/Hong Kong/WF10/1999(H9N2) and A/shorebird/Delaware/172/2006 (H16N3) (the wild‐type [WT] are henceforth known as H9 WT and H16 WT, respectively).

The highest SHAP values for α2,6‐linked sialic acid binding preference are listed in Table 2. Three iterations of site directed mutagenesis were performed. First, positions with the highest magnitude SHAP values for each HA were evaluated (position L226Q for H9 and K160A for H16 WT). Second, positions that had two relatively high SHAP values at the same position (suggesting the specific amino acid substitution) and were within two nucleotide changes of WT (G145S and Q227G for H9 WT and R227A for H16 WT) were evaluated. Finally, substitutions at a series of positions with high SHAP values near the receptor binding site were evaluated for potential additive effect on binding preference (G159S, K160A, G222K, R227A, and S228G).

**TABLE 2:** Highest magnitude SHAP values from CNN predictions of α2,6‐linked sialic acid binding preference on two IAV strains.

A/guinea fowl/Hong Kong/WF10/1999(H9N2)		A/shorebird/Delaware/172/2006(H16N3)	
Position	SHAP	Position	SHAP
226Q	0.2206	160A	0.0665
226L	0.0682	327Q	0.0589
145G	0.0221	328N	0.0535
531F	0.0173	327E	0.0487
227Q	0.0133	227R	0.0449
189A	0.0127	532L	0.0431
196V	0.0115	122E	0.0391
508S	0.0113	222G	0.0386
227X	0.0112	500Q	0.0381
63D	0.0107	222K	0.0341
227G	0.0101	159S	0.0341
63T	0.0096	164V	0.0336
508K	0.0091	50K	0.0324
24T	0.0090	227A	0.0320
196K	0.0084	527L	0.0312
219P	0.0082	228G	0.0287
523—	0.0081	489E	0.0279
145S	0.0075	327S	0.0273
262G	0.0074	431L	0.0268
188T	0.0067	454T	0.0256

*Note:* HA positions are listed using H3 numbering. Positions tested in the first round of site directed mutagenesis are written in orange, the second in blue, and third in green. A higher SHAP value implies that the presence or absence of a specific amino acid at that position contributes more to the prediction generated by the model. For example, for the H9N2 sample, whether or not there was a glutamine (Q) at position 226, it was highly informative to the prediction made by the model, while a whether or not there was leucine (L) at position 226 had less of an impact on the prediction generated. This may suggest that a glutamine is more associated with binding to one of the receptor types.

Results of binding analyses after these HA amino acid substitutions were performed are presented in Table 3. The L226Q substitution was the only change to impact H9 receptor binding; terminal sialic acid conformation affinity was entirely reversed. No switches in binding preference from α2,6‐ to α2,3‐linked sialic acids were observed for H16 with these substitutions, though some changes did diminish preference for both α2,3‐ and α2,6‐linked sialic acid receptors (R227A, G222K, as well as the 2, 3, and 4 amino acid substitution H16s). Additionally, H16 S228G agglutinated RBCs but did not agglutinate RBCs after resialylation with α2,3‐ or α2,6‐linked sialic acid receptors. In combination with the four other amino acid changes, S228G resulted in no agglutination of RBCs.

**TABLE 3:** Impact of site‐directed mutagenesis of H9 and H16 viruses on sialic acid binding.

Site‐directed mutagenesis Round 1: change at highest SHAP value							
Subtype	Residue numbering		Predicted probability	RBC	dRBC	3‐RBC	6‐RBC
	160	226					
H9 WT	F	L	0.6665	64	0	0	64
H9 L226Q	F	Q	0.0000	64	0	64	0
H16 WT	K	Q	0.8937	128	0	64	128
H16 K160A	A	Q	0.0837	128	0	64	128

Site‐directed mutagenesis Round 2: change at positions with high SHAP value with a suggested direction and within two nucleotide changes							
Subtype	Residue numbering		Predicted probability	RBC	dRBC	3‐RBC	6‐RBC
	145	227					
H9 WT	G	Q	0.6665	128	0	0	128
H9 G145S	S	Q	0.6571	128	0	0	128
H9 Q227G	G	G	0.0000	128	0	0	128
H16 WT	A	R	0.8937	128	0	64	128
H16 R227A	A	A	0.1730	128	0	16	64

Site‐directed mutagenesis Round 3: additive changes with high SHAP values										
Subtype	Residue numbering					Predicted probability	RBC	dRBC	3‐RBC	6‐RBC
	159	160	222	227	228					
H16 WT	G	K	G	R	S	0.8937	128	0	32	128
H16 G159S	S	K	G	R	S	0.4387	128	0	32	128
H16 G222K	G	K	K	R	S	0.2156	128	0	4	64
H16 S228G	G	K	G	R	G	0.5847	128	0	0	0
H16 K160A + R227A	G	A	G	A	S	0.0023	128	0	2	64
H16 K160A + R227A + G222K	G	A	K	A	S	0.0000	128	0	0	64
H16 K160A + R227A + G222K + G159S	S	A	K	A	S	0.0000	128	0	0	64
H16 K160A + R227A + G222K + G159S + S228G	S	A	K	A	G	0.0000	0	0	0	0

*Note:* Hemagglutination unit titers are reported for untreated turkey red blood cells (RBC), desialylated red blood cells (dRBC), red blood cells resialylated by α2,3‐sialyltransferase (3‐RBC), and red blood cells resialylated by α2,6‐sialyltransferase (6‐RBC). Residues are colored by Clustal X convention.

### 3.3 Impact of Missing Sequence Data on Predictions

**FIGURE 4:**
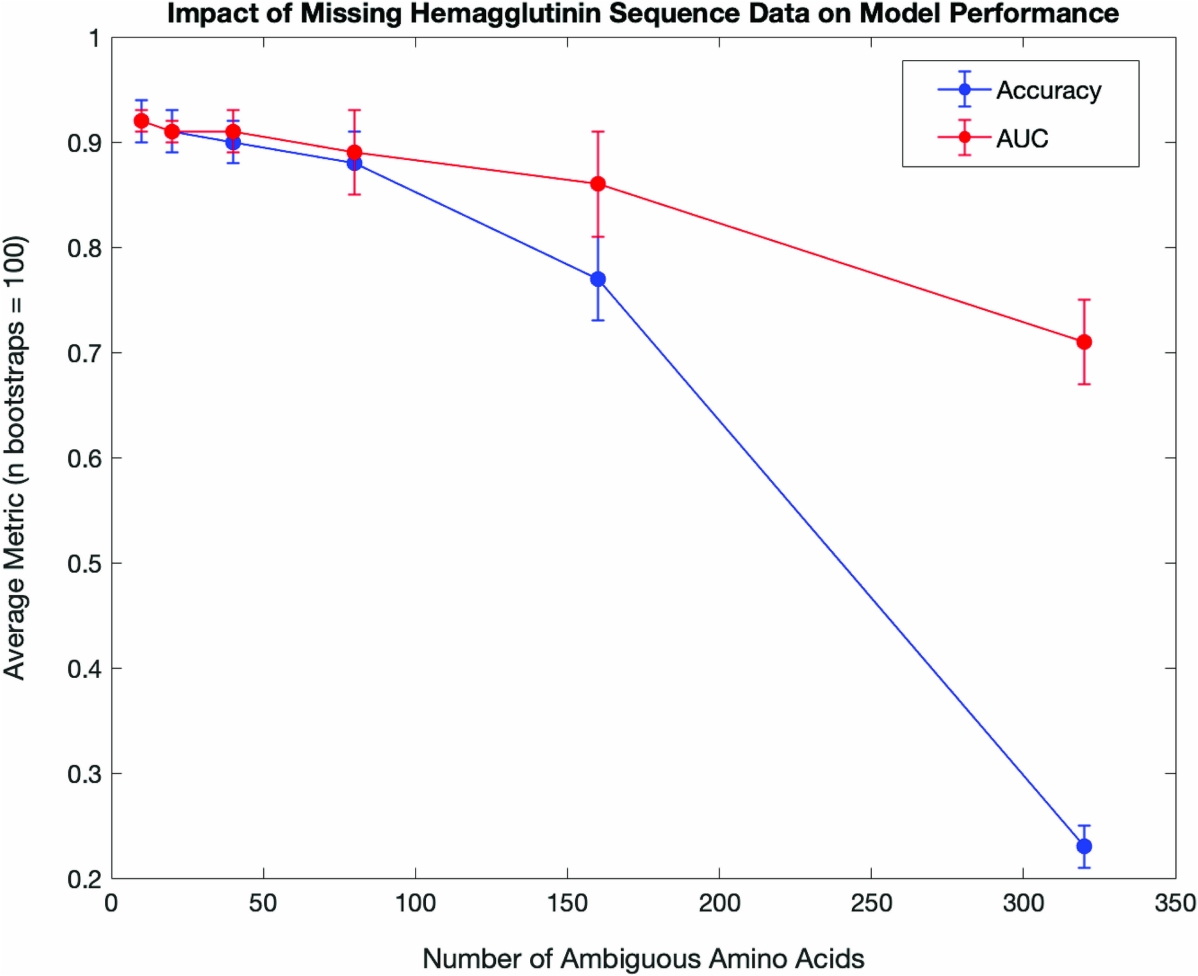
Convolutional neural network test predictions on sequences with missing data. A length of missing data was coded randomly into each test data sequence prior to model prediction. Bootstraps (100) were performed for each length of missing amino acids. The mean and standard deviation of the area under the receiver operating characteristic curve (AUC) and accuracy were recorded.

**FIGURE 5:**
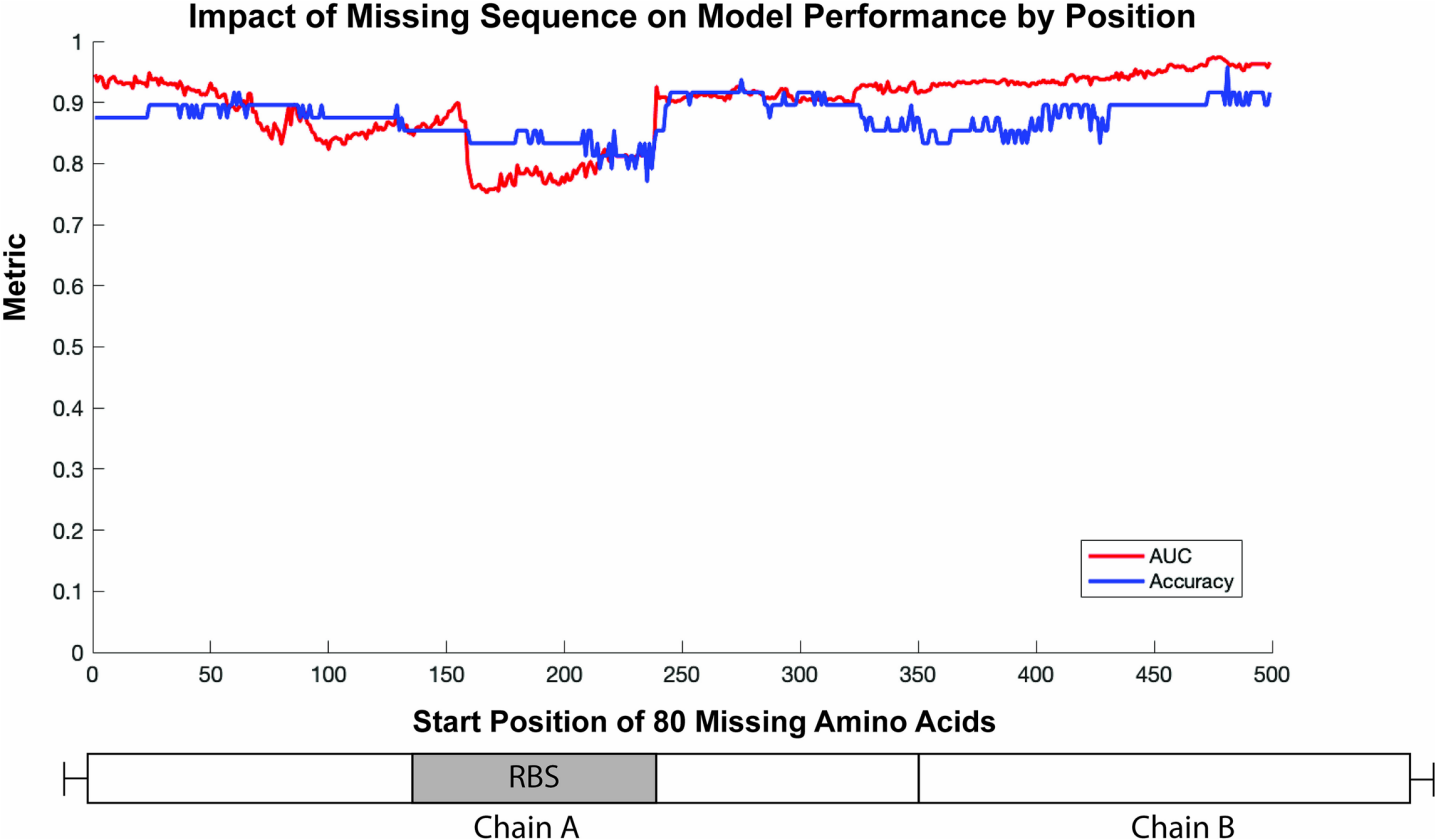
Convolutional neural network predictions on sequences with missing data by position. A length of missing data was coded into each possible start position of the test data prior to model prediction. The area under the receiver operating characteristic curve (AUC) and accuracy were recorded. A simple illustration of hemagglutinin is below the plot to give context for the missing data start position. Chains A and B are separated by the protease cleavage site. The receptor binding site (RBS) includes the 130‐Loop, 150‐Loop, 190‐Helix, and 220‐Loop.

To understand how well this model tolerated missing sequence data, varying lengths of the test sequences were artificially coded as missing (ambiguous amino acid “X”), and test predictions were generated. Predictions for test sequences with 40 consecutive missing amino acids showed an AUC of 0.91 and accuracy of 0.90 (Figure 4). Performance rapidly deteriorated after more than 80 missing amino acids per sequence. To understand where missing sequence data most impacted model performance, predictions were generated on the test data with 80 missing amino acids at each possible starting position in the sequence. Missing data on either end of the HA gene had little impact on the AUC and accuracy (Figure 5). There was a notable dip in performance (AUC between 0.7 and 0.8, accuracy between 0.7 and 0.85) where the missing sequence removes part or all of the receptor binding site structures.

### 3.4 Prediction on Novel HA Subtypes

**FIGURE 6:**
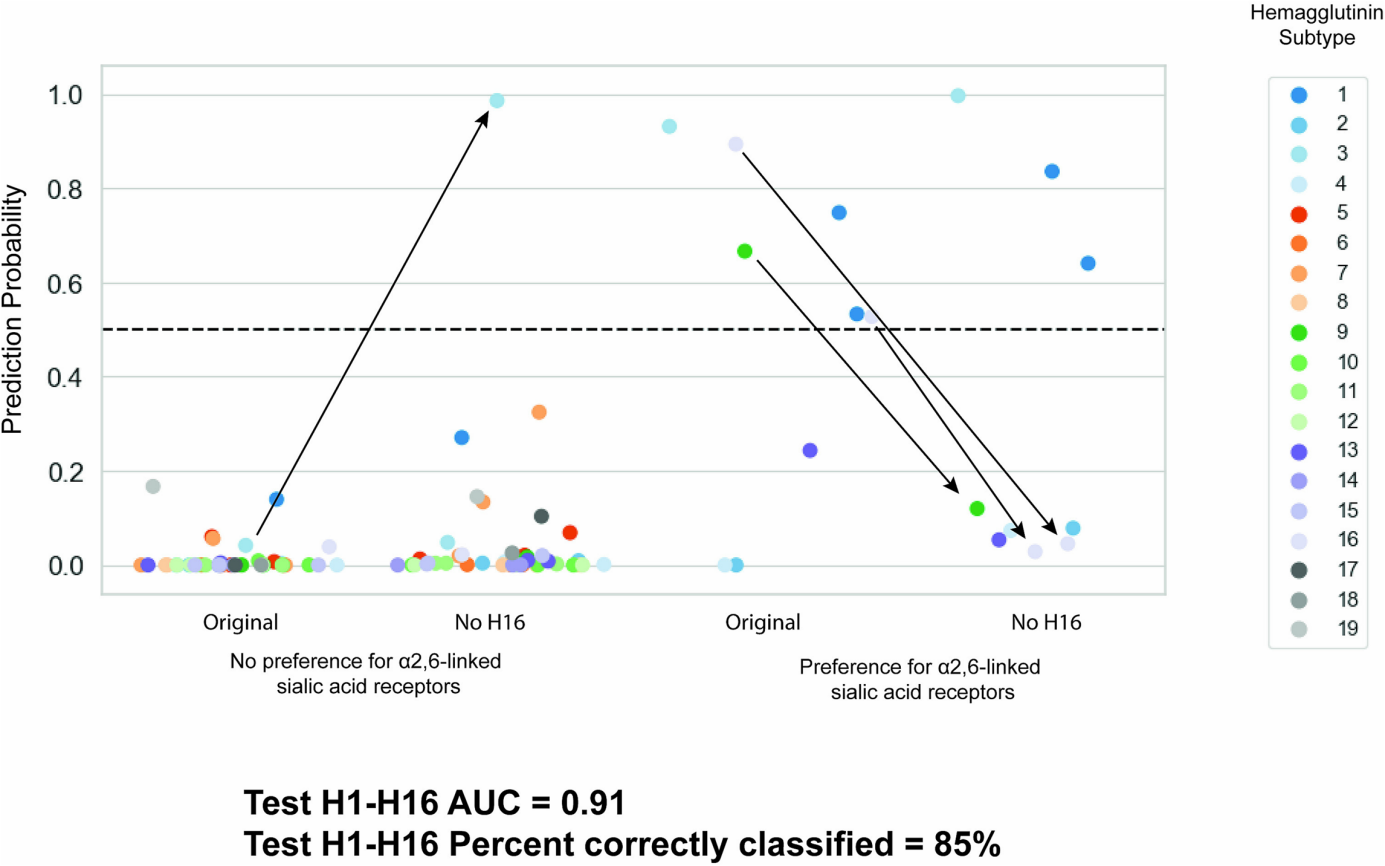
Probabilities predicted from an independent test of a convolutional neural network trained without samples of subtype H16 to classify hemagglutinin sequences by α2,6‐linked sialic acid binding preference. Arrows indicate sample predictions that changed class after omission of H16 in the training data. The test metrics do not include predictions on H17, H18, or H19.

With all H16 samples removed from all parts of model training (base model, binding data, and validation data for early stopping), a CNN generated predictions on the independent test dataset with an AUC of 0.91 and accuracy of 0.85 (Figure 6). All three of the H16 test samples were classed as not preferring α2,6‐linked sialic acid receptors, thus two were incorrectly classified. In addition to changes in H16 classification, the model trained without H16 training examples also failed to correctly classify A/camel/Mongolia/335/2012(H3N8) and A/Guinea Fowl/Hong Kong/WF10/1999(H9N2). On the more novel, non‐sialic acid binding subtypes of H17, H18, and H19, both the fully trained CNN and the CNN lacking H16 classified these samples as having no preference for α2,6‐linked sialic acid receptors with a probability below 0.2.

## 4 Discussion

### 4.1 General Model Performance

In this study, we developed a machine learning model trained to predict the sialic acid conformation preference of IAVs given the HA sequence. Our tests on an independent dataset showed that 94% of the test predictions were correct and the test AUC was 0.93. Dissecting the model test predictions reveals that there were no false positive predictions, and the samples with no preference for α2,6‐linked sialic acids were more certain (closer to the true label) than those with a true preference for α2,6‐linked sialic acids. This can be attributed to the class imbalance of the training data. Across all IAVs, preference for α2,6‐linked sialic acids is a more rare phenotype both in nature and in the available training data. While class‐weighting was applied to help reduce biases, it did not fully mitigate the effect of the imbalance. Thus, the model tended towards a prediction of no preference for α2,6‐linked sialic acids. A closer look at the incorrectly classified samples revealed that the model misclassified some uncommon subtype and binding preference pairings. Two mammalian‐origin IAVs of subtypes H2 and H4 were incorrectly classified as not preferring α2,6‐linked sialic acids; these HA types are more commonly found in avian hosts, which may signal to misclassification due to this anomaly. The other incorrectly classified sample was a gull‐derived H13 virus with a preference for α2,6‐linked sialic acids. H13 influenza viruses are most commonly detected among Charadriiforms, especially gulls, and evidence of H13 influenza viruses that bind to α2,6‐linked sialic acid receptors is limited [16], so this too represents an anomalous example that was understandably misclassified. These findings signal to room for improvement in the diversity of samples in the training dataset.

One future direction for increasing the available sample size is the development of higher throughput assays for determining IAV receptor binding specificity. In the absence of additional binding data, use of generative algorithms to create synthetic datapoints is an alternative approach worth exploring. One such example are generative adversarial networks; this approach pits two neural networks against each other, one to generate fake datapoints based on the distribution of a real dataset, and one to discriminate between real and fake datapoints that are generated. In doing so, realistic, synthetic data are produced to help improve model performance with less risk of overfitting a small number of training samples. Alternatively, repositories to share paired viral genotype and phenotype data would allow for a more concerted effort among influenza researchers, enable large‐scale analyses with more sophisticated *in silico* approaches, and overcome limitations in resources and time that prevent individual labs from achieving sample sizes that are great enough for large‐scale data analyses.

### 4.2 Model‐Derived Amino Acid Importance

To understand what features of the amino acid sequences most impacted prediction of sialic acid binding preference, we calculated SHAP values. Globally, the highest mean SHAP values were identified in the HA1 region of the protein. The highest magnitude feature was at position 226. A Q226 L substitution is often regarded as pivotal to change avian IAV receptor binding preference and has previously been shown to confer this change across multiple subtypes [17, 18, 19]. Overall, this suggests that the model does recognize meaningful features.

Taking this further, we calculated the SHAP values of H9 and H16 test sequences that exhibited affinity for α2,6‐linked sialic acid receptors. The highest magnitude SHAP value, by some margin, for H9 was 226Q. Indeed, a substitution of L226Q completely reversed the binding behavior of the H9 pseudovirus. No other H9 substitutions were found to impact binding in this way. For H16, there were not the same type of standout substitutions as H9 Q226L. We tested a variety of substitutions informed by the highest SHAP values for positions in the receptor binding structures, but no switch from α2,6‐ to α2,3‐linked sialic acid preference was observed. Some of the amino acid changes tested did affect the binding phenotype, including one position that obliterated binding to both α2,6‐ and α2,3‐linked sialic acids, but still retained the ability to hemagglutinate sialylated RBCs. We suspect that this reflects a binding preference for α2,8‐linked sialic acid terminated receptors, as these receptors are also removed during the desialylation. Additionally, two of the three H16 proteins (A/mallard/Gurjev/785/83 (H16N3) and A/black‐headed gull/Sweden/2/99 (H16N3)) that have binding data available on CFG showed a high affinity for α2,8‐linked sialic acids. Still, future work to understand if H16 viruses have a broader affinity for other sialic acid conformations or if these observations are an artifact of laboratory manipulation is warranted.

Looking more closely at positions identified in the H16 substitution search, there is a dichotomy in the residues present at position 160 for IAVs that prefer α2,6‐ or α2,3‐linked sialic acids. Among IAVs that bind α2,3‐linked sialic acids in our training dataset, 30% of the samples have the A160 amino acid variant, while 5% have the K160 amino acid variant. On the other hand, for the IAVs that bind α2,6‐linked sialic acids, 4% have the A160 amino acid variant, while 32% have the K160 amino acid variant. Generally, K160 is more common among mammalian H3 and avian H13 or H16 viruses. Changes at position 160 that have previously been shown to impact sialic acid preference were related to loss of N‐linked glycosylation at N158 by a change to position 160 [20]. Interestingly, combinatorial substitutions at positions 160 and 227 have also been shown to influence binding preference of H5 viruses [21, 22]. Given the previous data that cites similar positions as were identified through this modeling, it is possible that the model is currently assigning classes based on subtypes with receptor binding site sequence similarities, but the training dataset is not yet at a state to reflect important differences between H16 viruses and H3 or H5 viruses that bind α2,6‐linked sialic acids.

Another explanation for why we were unable to identify changes that shifted receptor binding preference from α2,6‐ to α2,3‐linked sialic acids is that methods beyond SHAP values might be useful for assigning amino acid importance. Studies that use CNNs often visualize the convolutional filters that pick up patterns in the input data to aid interpretation of the decision‐making process [23]. In the more typical application of CNN modeling for image classification, convolutional filters can be mapped to an input image to highlight regions of the image that had a pattern picked up by the filter. However, the interpretation of such filters is more complicated for a classification problem, such as this sequence‐based problem, that is not image‐based.

### 4.3 Evaluation of Model Limitations

Finally, other limits of the model and approach were probed by testing the impact of missing sequence data and testing prediction on novel subtypes, respectively. For the former, different lengths of missing amino acids were coded into the test datapoints at random to mimic prediction on low coverage sequences. This analysis showed that sequences with up to 40 missing amino acids resulted in minimal impact on prediction performance, with average performance metrics above 0.9. Even predictions on sequences with 80 missing amino acids are well above random chance. Thereafter, however the performance dips considerably. This suggests that this modeling approach is tolerant to a reasonable degree (~10%) of missing data. The location of the missing sequence data was an important determinant of the loss in performance. We found that missing data at the ends of the HA gene had less impact on performance than missing data that obscured the receptor binding structures. This demonstrates that accurate predictions could be made on some low coverage sequences depending on the positions that are impacted and can also serve as further evidence that the model pulls predictions from meaningful features of the input sequence. To assess prediction performance on novel subtypes, the same CNN modeling approach was applied without representation of H16 sequences in the base model and training data. The test performance on H16 takes an understandable dip when compared to the model trained with H16 samples. This represents a model limitation that the approach requires future refinement before this modeling is used on novel subtypes. Interestingly, prediction of other subtypes was also impacted by the exclusion of H16 training data, namely A/camel/Mongolia/335/2012 (H3N8) and A/Guinea Fowl/Hong Kong/WF10/1999 (H9N2). This demonstrates the transferability of information related to binding behavior across subtypes and underscores the importance of a heterosubtypic approach to decoding the HA receptor binding structures.

### 4.4 Limitations and Future Directions

There are several limitations to this model beyond the aforementioned sample size and diversity constraints of the training data. Given that protein alignment was used to preprocess the sequences for input, this model does not handle novel insertions. Since this model only uses virus sequence data to generate a binding prediction, differences in binding behavior of different virus or protein preparation types are not accounted for in prediction generation; these may include the effects that post‐translational modifications, like glycosylation, may have on binding activity, which have been previously observed [24]. Future studies might also benefit from including input features beyond the linear amino acid sequences used in this study. Other studies that have applied machine learning approaches to prediction of IAV host or antigenicity have used inputs that incorporate protein properties in the input, such as secondary structures and van der Waals volume [25, 26]. Such an approach may overcome some of the limitations of the CNN approach used here, which is more tuned to localized relationships in the input sequence. Finally, this model cannot be used for prediction of IAV binding beyond α2,3‐ or α2,6‐linked sialic acid receptor preference. Recent studies have showed a shift in affinity from sialylated to nonsialylated glycans over time for human‐adapted seasonal IAVs due to antigenic pressure [27, 28]. Additionally, multiple subtypes of IAVs (H2, H17, H18, and H19) have recently been found to use MHCII proteins as receptors [29, 30, 31], and bat‐derived H9N2 may also share this phenotype given similarities to the H19 receptor binding site [32]. While there is currently not sufficient publicly available data to model HA binding to these alternative receptors, this is an important area for future research. With this model, we were able to, to a limited degree, identify amino acid changes that impacted H16 receptor binding, a subtype for which the receptor binding sites have not been well studied for phenotypic effects. While we hypothesize some of the changes made inadvertently increased H16 affinity to α2,8‐linked sialic acid, ascertaining preference for other sialic acid conformations was outside of the scope of this study.

Ultimately, this study presents an in silico approach to sialic acid conformation‐preference prediction of IAVs in a way that our tests suggest are generalizable to unseen sequences and draw predictions from regions with known association to receptor binding specificity. Future validation and refinement of the modeling approach used in this study is needed before it is field‐ready for critical decision making and wide use. Areas for improvement upon the modeling include expansion of the training dataset, in particular, to balance the α2,3‐sialic acid binding bias, to improve prediction performance and further interrogation into how to decode these types of models in a way that allows for prediction of amino acid substitutions that change receptor binding preferences. With improvements, models of this sort could be useful additions to the Tool for Pandemic Risk Assessment and Influenza Risk Assessment Tool protocols for identifying viruses showing signs of mammalian adaptation and enhanced transmissibility in humans.

## Author Contributions

**Laura K. Borkenhagen:** conceptualization, writing – original draft, funding acquisition, data curation, formal analysis. **Jonathan A. Runstadler:** conceptualization, funding acquisition, writing – review and editing, supervision.

## Conflicts of Interest

The authors declare no conflicts of interest.

### Peer Review

The peer review history for this article is available at https://www.webofscience.com/api/gateway/wos/peer‐review/10.1111/irv.70044.

## Supporting information


**Figure S1** Phylogenetic tree of training (Consortium for Functional Glycomics in blue, other source in orange) and testing (green) hemagglutinin (subtypes denoted) amino acid sequences for the α2,6‐linked sialic acid receptor binding model. This tree was generated using MEGAX (https://www.megasoftware.net/). The branches were calculated with the neighbor‐joining method under the Poisson model with bootstrapping (*N* = 1000), substitution rates set in Gamma distribution (α = 1.0), and gaps treated in pairwise deletion. This tree serves as visual of the distances between the training and testing datapoints and is not meant for inference of evolutionary relationships.
**Table S1.** Sources of viruses used in this study.
**Table S2.** Glycan pairings from CFG microarrays. Glycans are written using standard linear nomenclature (see National Center for Biotechnology Information for details [1]). Sp# indicates the spacer used to immobilize the glycan (see Grant et al. for details [2]).
**Table S3.** Convolutional neural network parameters and hyperparameters.
**Table S4.** Primers for amplification of hemagglutinin for assembly into a pcDNA3.1(+) vector. All sequences are written 5′ to 3′. Sequence that anneals to the vector is italicized, NheI and BamHI restriction sites are underlined, and sequence that anneals to the HA insert is bolded.
**Table S5.** Primers used for site directed mutagenesis of hemagglutinin. Primers were designed using NEBaseChanger with parsimony. The base changes for each primer set are in bold.

**Data S1** FASTA file of all sequences used in binding modeling.

**Data S2** Sialic acid preference labels of all sequences used in binding modeling. Label “0” indicates no preference for α2,6‐linked sialic acid, and “1” indicates preference for α2,6‐linked sialic acid.

## Data Availability

Sequences and labels of all binding data can be found in the supporting information (Data S1 and Data S2, respectively). The models and the code used to produce them are available from Bitbucket Git repository: https://bitbucket.org/borkenhagen‐workspace/influenza_binding.
