# Supplementary material for: Examining the Influenza A Virus Sialic Acid Binding Preference Predictions of a Sequence‐Based Convolutional Neural Network
Source: Influenza Other Respir Viruses. 2024 Dec 11;18(12):e70044. doi: 10.1111/irv.70044 (PMC11634464; doi:10.1111/irv.70044)
Supplement: Supplementary file 1 — Figure S1 Phylogenetic tree of training (Consortium for Functional Glycomics in blue, other source in orange) and testing (green) hemagglutinin (subtypes denoted) amino acid sequences for the α2,6‐linked sialic acid receptor binding model. This tree was generated using MEGAX (https://www.megasoftware.net/). The branches were calculated with the neighbor‐joining method under the Poisson model with bootstrapping (N = 1000), substitution rates set in Gamma distribution (α = 1.0), and gaps treated in pairwise deletion. This tree serves as visual of the distances between the training and testing datapoints and is not meant for inference of evolutionary relationships. Table S1. Sources of viruses used in this study. Table S2. Glycan pairings from CFG microarrays. Glycans are written using standard linear nomenclature (see National Center for Biotechnology Information for details [1]). Sp# indicates the spacer used to immobilize the glycan (see Grant et al. for details [2]). Table S3. Convolutional neural network parameters and hyperparameters. Table S4. Primers for amplification of hemagglutinin for assembly into a pcDNA3.1(+) vector. All sequences are written 5′ to 3′. Sequence that anneals to the vector is italicized, NheI and BamHI restriction sites are underlined, and sequence that anneals to the HA insert is bolded. Table S5. Primers used for site directed mutagenesis of hemagglutinin. Primers were designed using NEBaseChanger with parsimony. The base changes for each primer set are in bold. [file IRV-18-e70044-s001.docx]

**Examining the Influenza A Virus Sialic Acid Binding Preference Predictions of a Sequence-Based Convolutional Neural Network**

**Supporting Information**


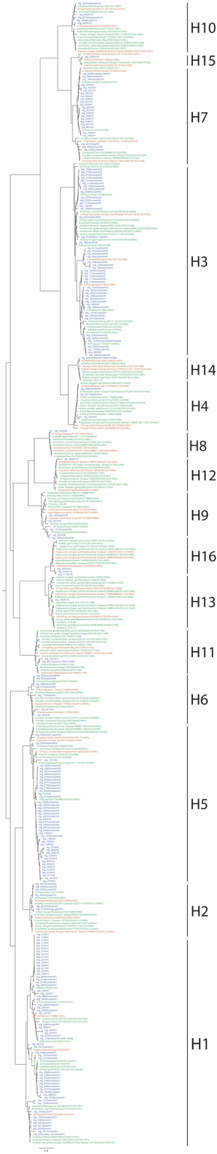
**Figure S1. Phylogenetic tree of training (CFG in blue, other source in orange) and testing (green) hemagglutinin (subtypes denoted) amino acid sequences for the α2,6-linked sialic acid receptor binding model.** This tree was generated using MEGAX (<https://www.megasoftware.net/>). The branches were calculated with the neighbor-joining method under the Poisson model with bootstrapping (N=1000), substitution rates set in Gamma distribution (α = 1.0), and gaps treated in pairwise deletion. This tree serves as visual of the distances between the training and testing datapoints and is not meant for inference of evolutionary relationships.

**Table S1. Sources of viruses used in this study.**

| **Strain Name** | **Source** | **Use** |
| --- | --- | --- |
| A/swine/Jamesburg/1942(H1N1) | Dr. Lisa Kercher | Test dataset |
| A/Taiwan/1/1986(H1N1) | Dr. Lisa Kercher | Test dataset |
| A/mallard/Chile/C4079/2015(H1N1) | Dr. Stacey Schultz-Cherry | Test dataset |
| A/American green-winged teal/Interior Alaska/11BM03165/2011(H2N9) | Runstadler Lab | Test dataset |
| A/mallard/Netherlands/5/1999(H2N9) | BEI Resources, NIAID, NIH | Test dataset |
| A/swine/Missouri/4296424/2006(H2N3) | Dr. Jürgen Richt | Test dataset |
| A/Philippines/2/1982(H3N2) | BEI Resources, NIAID, NIH | Test dataset |
| A/American green-winged teal/Alaska/19TL00145/2019(H3N8) | Runstadler Lab | Test dataset |
| A/camel/Mongolia/335/2012(H3N8) | BEI Resources, NIAID, NIH | Test dataset |
| A/swine/Missouri/A01727926/2015(H4N6) | Dr. Phillip Gauger | Test dataset |
| A/quail/California/D113023808/2012 (H4N2) | GenScript | Test dataset |
| A/king eider/Alaska/44068-067/2006(H4N7) | USGS | Test dataset |
| A/chicken/Texas/167280-4/2002(H5N3) | Dr. Ralph Tripp | Test dataset |
| A/fancy chicken/Netherlands/FAV-0033/2021(H5N1) | GenScript | Test dataset |
| A/yellow-billed teal/Chile/C24042/2017(H5N3) | Dr. Stacey Schultz-Cherry | Test dataset |
| A/goose/Interior Alaska/11PG00149/2011(H6N1) | Runstadler Lab | Test dataset |
| A/duck/Eastern China/11/2009(H6N6) | SinoBiological | Test dataset |
| A/shearwater/Australia/1/1973(H6N5) | BEI Resources, NIAID, NIH | Test dataset |
| A/mallard/Southcentral Alaska/15ML00267/2015(H7N3) | Runstadler Lab | Test dataset |
| A/mallard/Netherlands/12/2000(H7N3) | BEI Resources, NIAID, NIH | Test dataset |
| A/domestic cat/New York/WVDL-14/2016(H7N2) | Dr. Yoshihiro Kawaoka | Test dataset |
| A/northern pintail/Interior Alaska/9BM11643/2009(H8N4) | Runstadler Lab | Test dataset |
| A/mallard/Ohio/16OS0841/2016(H8N4) | Dr. Jaqueline Nolting | Test dataset |
| A/turkey/Ontario/6118/1968(H8N4) | BEI Resources, NIAID, NIH | Test dataset |
| A/gull/Massachusetts/13JR00943/2013(H9N1) | Runstadler Lab | Test dataset |
| A/shorebird/Delaware Bay/127/2003(H9N2) | BEI Resources, NIAID, NIH | Test dataset |
| A/Guinea Fowl/Hong Kong/WF10/1999(H9N2) | Dr. Anice Lowen | Test dataset |
| A/green-winged teal/Southcentral Alaska/18MB01702/2018(H10N7) | Runstadler Lab | Test dataset |
| A/chicken/Germany/N/1949 (H10N7) | BEI Resources, NIAID, NIH | Test dataset |
| A/seal/Netherlands/P14-221/2014(H10N7) | Dr. Lisa Kercher | Test dataset |
| A/least sandpiper/Southcentral Alaska/18MB01147/2018(H11N9) | Runstadler Lab | Test dataset |
| A/laughing gull/Delaware Bay/94/1995(H11N2) | BEI Resources, NIAID, NIH | Test dataset |
| A/shoveler/Netherlands/18/1999(H11N9) | BEI Resources, NIAID, NIH | Test dataset |
| A/mallard/Interior Alaska/11BM01009/2011(H12N5) | Runstadler Lab | Test dataset |
| A/mallard/Sweden/86/2003 (H12N5) | BEI Resources, NIAID, NIH | Test dataset |
| A/yellow-billed pintail/Chile/C14830/2016 (H12N5) | Dr. Stacey Schultz-Cherry | Test dataset |
| A/ring-billed gull/Massachusetts/13DC30736/2013 (H13N8) | Runstadler Lab | Test dataset |
| A/herring gull/Massachusetts/A00080255/2006(H13N2) | Runstadler Lab | Test dataset |
| A/gull/Cordova/17MB03606/2017(H13N2) | Runstadler Lab | Test dataset |
| A/mallard/Gurjev/263/1982(H14N5) | BEI Resources, NIAID, NIH | Test dataset |
| A/long-tailed duck/Wisconsin/10OS3912/2010(H14N6) | Dr. Jaqueline Nolting | Test dataset |
| A/blue-winged teal/Guatemala/CIP049-H123-13/2014(H14N3) | Dr. Daniel Perez | Test dataset |
| A/shearwater/Australia/2576/1979(H15N9) | BEI Resources, NIAID, NIH | Test dataset |
| A/mallard/Novomychalivka/2-23-12/2010(H15N7) | GenScript | Test dataset |
| A/duck/AUS/341/1983(H15N8) | SinoBioogical | Test dataset |
| A/glaucous-winged gull/Southcentral Alaska/11JR02272/2011(H16N3) | Runstadler Lab | Test dataset |
| A/shorebird/Delaware/172/2006(H16N3) | BEI Resources, NIAID, NIH | Test dataset |
| A/gull/Massachusetts/13WP00539/2013(H16N3) | Runstadler Lab | Test dataset |
| A/kelp gull/Chile/C8939/2016(H13N2) | Dr. Stacey Schultz-Cherry | Train dataset |
| A/glaucous gull/Alaska/44198-027/2006(H16N3) | USGS | Train dataset |
| A/duck/Alaska/11PG0459/2011(H2N3) | Runstadler Lab | Train dataset |
| A/human/Baltimore/0244/2017(H3N2) | Dr. Andrew Pekosz | Train dataset |
| A/gull/Massachusetts/14WP00016/2014(H4N4) | Runstadler Lab | Train dataset |
| A/glaucous-winged gull/Southcentral Alaska/13MB02558/2013(H16N3) | Runstadler Lab | Train dataset |
| A/ruddy turnstone/New Jersey/UGAI16-2139/2016(H6N5) | Dr. Rebecca Poulson | Train dataset |
| A/yellow-billed teal/Chile/C25814/2017(H7N6) | Dr. Stacey Schultz-Cherry | Train dataset |
| A/northern pintail/Interior Alaska/8MP0547/2008(H8N4) | Runstadler Lab | Train dataset |
| A/swine/Ohio/16TOSU5421/2016(H1N2) | Dr. Jaqueline Nolting | Train dataset |
| A/shorebird/Delaware Bay/338/2009(H10N1) | BEI Resources, NIAID, NIH | Train dataset |
| A/shorebird/Delaware Bay/216/1999(H11N2) | BEI Resources, NIAID, NIH | Train dataset |
| A/mallard/Wisconsin/4218/2009(H12N5) | BEI Resources, NIAID, NIH | Train dataset |
| A/glaucous-winged gull/Southcentral Alaska/12NH01285/2012(H16N3) | Runstadler Lab | Train dataset |
| A/kelp gull/Chile/C10791/2016(H16N3) | Dr. Stacey Schultz-Cherry | Train dataset |
| A/ring-billed gull/Massachusetts/12DC00060/2012(H13N6) | Runstadler Lab | Train dataset |
| A/blue-winged teal/Ohio/18OS1695/2018(H14N5) | Dr. Jaqueline Nolting | Train dataset |
| A/herring gull/Massachusetts/A00648685/2008(H13N2) | Runstadler Lab | Train dataset |
| A/mallard/Ohio/16OS0869/2016(H7N3) | Dr. Jacqueline Nolting | Train dataset |
| A/Brisbane/59/2007(H1N1) | BEI Resources, NIAID, NIH | Train dataset |
| A/mallard/Maine/A00198555/2007(H1N1) | Runstadler Lab | Train dataset |
| A/mallard/Republic of Georgia/4/2010(H1N1) | BEI Resources, NIAID, NIH | Train dataset |
| A/mallard/Interior Alaska/12ML00957/2014(H1N1) | Runstadler Lab | Train dataset |
| A/Baltimore/R0252/2018(H1N1) | Dr. Andrew Pekosz | Train dataset |
| A/Taipei/0032/2016(H1N1) | Dr. Andrew Pekosz | Train dataset |
| A/swine/Ohio/19TOSU4462/2019(H1N2) | Dr. Jacqueline Nolting | Train dataset |
| A/Fort Monmouth/1/1947(H1N1) | BEI Resources, NIAID, NIH | Train dataset |
| A/NWS/1933(H1N1) | BEI Resources, NIAID, NIH | Train dataset |
| A/Tennessee/F5029/2014(H1N1) | Dr. Charles Russell | Train dataset |
| A/Georgia/F32551/2012(H1N1)pdm09 | BEI Resources, NIAID, NIH | Train dataset |
| A/ruddy turnstone/South Carolina/UGAI17-4979/2017(H2N5) | Dr. Rebecca Poulson | Train dataset |
| A/American Black Duck/Maine/A00090256/2007(H3N2) | Runstadler Lab | Train dataset |
| A/American green-winged teal/Interior Alaska/9BM12356/2009(H3N8) | Runstadler Lab | Train dataset |
| A/long-tailed duck/Wisconsin/16OS4632/2016(H3N8) | Runstadler Lab | Train dataset |
| A/mallard/Interior_Alaska/9BM12326/2009(H3N8) | Runstadler Lab | Train dataset |
| A/swine/Mexico/SG1444/2011(H3N2) | BEI Resources, NIAID, NIH | Train dataset |
| A/Hong Kong/1/1968(H3N2) | Dr. Lisa Kercher | Train dataset |
| A/canine/Illinois/41915/2015(H3N2) | Dr. Colin Parrish | Train dataset |
| A/blue-winged teal/Illinois/10OS1546/2010(H3N6) | BEI Resources, NIAID, NIH | Train dataset |
| A/blue-winged teal/Iowa/10OS2411/2010(H3N8) | BEI Resources, NIAID, NIH | Train dataset |
| A/duck/Ukraine/1963(H3N8) | BEI Resources, NIAID, NIH | Train dataset |
| A/Sydney/5/1997(H3N2) | BEI Resources, NIAID, NIH | Train dataset |
| A/Rochester/0090/2013(H3N2) | Dr. David Topham | Train dataset |
| A/swine/Kansas/2014-102/2014(H3N2) | Dr. Wenjun Ma | Train dataset |
| A/northern pintail/Alaska/19TL00162/2019(H3N8) | Runstadler Lab | Train dataset |
| A/Victoria/210/2009(H3N2) | Dr. Lisa Kercher | Train dataset |
| A/duck/Czechoslovakia/1956(H4N6) | BEI Resources, NIAID, NIH | Train dataset |
| A/northern pintail/Interior Alaska/10BM07040/2010(H4N6) | Runstadler Lab | Train dataset |
| A/American green-winged teal/Alaska/19TL00078/2019(H4N6) | Runstadler Lab | Train dataset |
| A/yellow-billed pintail/Chile/6/2013(H4N6) | Dr. Stacey Shultz-Cherry | Train dataset |
| A/blue-winged teal/Alberta/346/2007(H4N3) | BEI Resources, NIAID, NIH | Train dataset |
| A/blue-winged teal/Wisconsin/402/1983(H4N6) | BEI Resources, NIAID, NIH | Train dataset |
| A/red knot/Delaware/541/1988(H4N6) | BEI Resources, NIAID, NIH | Train dataset |
| A/blue-winged teal/Illinois/10OS1563/2010(H4N6) | BEI Resources, NIAID, NIH | Train dataset |
| A/glaucous-winged gull/Southcentral Alaska/17MB01587_1/2017(H4N7) | Runstadler Lab | Train dataset |
| A/turkey/Massachusetts/3740/1965(H6N2) | BEI Resources, NIAID, NIH | Train dataset |
| A/ruddy turnstone/New Jersey/UGAI14-1399/2014(H6N1) | Dr. Rebecca Poulson | Train dataset |
| A/ruddy turnstone/Delaware/892/2002(H6N1) | Dr. Mark Tompkins | Train dataset |
| A/ruddy turnstone/New Jersey/UGAI16-2120/2016(H6N8) | Dr. Rebecca Poulson | Train dataset |
| A/mallard/Alberta/203/1992(H6N5) | BEI Resources, NIAID, NIH | Train dataset |
| A/swine/Guangdong/K6/2010(H6N6) | Dr. Xiu-Feng Wan | Train dataset |
| A/mallard/Interior Alaska/10BM07066/2010(H7N3) | Runstadler Lab | Train dataset |
| A/ruddy turnstone/New Jersey/UGAI15-3254/2015(H7N3) | Dr. Rebecca Poulson | Train dataset |
| A/northern pintail/Alaska/UGAI16-3997/2016(H8N4) | Dr. Rebecca Poulson | Train dataset |
| A/ruddy turnstone/Virginia/2297/1988(H9N9) | BEI Resources, NIAID, NIH | Train dataset |
| A/turkey/Wisconsin/1/1966(H9N2) | BEI Resources, NIAID, NIH | Train dataset |
| A/shorebird/Delaware Bay/31/1996(H9N7) | BEI Resources, NIAID, NIH | Train dataset |
| A/common eider/Massachusetts/A00165665/2006(H10N2) | Runstadler Lab | Train dataset |
| A/mallard/Netherlands/1/2014(H10N7) | Dr. Lisa Kercher | Train dataset |
| A/mallard/Wisconsin/455/1979(H10N7) | BEI Resources, NIAID, NIH | Train dataset |
| A/shorebird/Delaware Bay/102/2000(H10N4) | BEI Resources, NIAID, NIH | Train dataset |
| A/mallard/Illinois/10OS4334/2010(H10N7) | BEI Resources, NIAID, NIH | Train dataset |
| A/blue-winged teal/Louisiana/UGAI14-430/2014(H10N3) | Dr. Rebecca Poulson | Train dataset |
| A/ruddy turnstone/New Jersey/UGAI16-1299/2016(H10N9) | Dr. Rebecca Poulson | Train dataset |
| A/environment/Maryland/17OS3341/2017(H10N3) | Dr. Jacqueline Nolting | Train dataset |
| A/duck/England/1956(H11N6) | BEI Resources, NIAID, NIH | Train dataset |
| A/lesser black-backed gull/Iceland/145/2010(H11N2) | BEI Resources, NIAID, NIH | Train dataset |
| A/mallard/Alberta/125/1999(H11N6) | BEI Resources, NIAID, NIH | Train dataset |
| A/yellow-billed teal/Chile/C8619/2016(H11N9) | Dr. Stacey Shultz-Cherry | Train dataset |
| A/ruddy turnstone/New Jersey/UGAI16-1474/2016(H11N9) | Dr. Jacqueline Nolting | Train dataset |
| A/common goldeneye/Wisconsin/18OS2949/2018(H11N9) | Dr. Jacqueline Nolting | Train dataset |
| A/ruddy turnstone/New Jersey/UGAI16-1492/2016(H11N5) | Dr. Rebecca Poulson | Train dataset |
| A/mallard/Maryland/16OS2559/2016(H12N5) | Dr. Jacqueline Nolting | Train dataset |
| A/ruddy turnstone/Delaware/328/2018(H12N5) | Dr. Lisa Kercher | Train dataset |
| A/mallard/Interior Alaska/12ML00678/2013(H12N5) | Runstadler Lab | Train dataset |
| A/northern pintail/Missouri/319/2009(H12N5) | BEI Resources, NIAID, NIH | Train dataset |
| A/UGAI14-1147/ruddy turnstone/New Jersey/2014(H12N4) | Dr. Rebecca Poulson | Train dataset |
| A/ruddy turnstone/South Carolina/UGAI18-1316/2018(H12N5) | Dr. Rebecca Poulson | Train dataset |
| A/American white pelican/Minnesota/Sg-0611/2008(H13N9) | Dr. Rebecca Poulson | Train dataset |
| A/glaucous-winged gull/Southcentral Alaska/15MB01429/2015(H13N6) | Runstadler Lab | Train dataset |
| A/glaucous-winged gull/Southcentral Alaska/15MB01632/2015(H13N6) | Runstadler Lab | Train dataset |
| A/gull/Maryland/704/1977(H13N6) | BEI Resources, NIAID, NIH | Train dataset |
| A/glaucous-winged gull/Southcentral Alaska/18MB00688/2018(H13N6) | Runstadler Lab | Train dataset |
| A/kelp gull/Chile/C27733/2017(H13N8) | Dr. Stacey Shultz-Cherry | Train dataset |
| A/environment/Chile/C20182/2016(H13N9) | Dr. Stacey Shultz-Cherry | Train dataset |
| A/blue-winged teal/Texas/UGAI15-6890/2015(H14N7) | Dr. Rebecca Poulson | Train dataset |
| A/mallard/Astrakhan/263/1982(H14N5) | BEI Resources, NIAID, NIH | Train dataset |
| A/northern shoveler/Mississippi/12OS456/2012(H14N2) | Dr. Jacqueline Nolting | Train dataset |
| A/brown-hooded gull/Chile/C8851/2016(H16N3) | Dr. Stacey Shultz-Cherry | Train dataset |
| A/glaucous-winged gull/Southcentral Alaska/16MB02941/2016(H16N3) | Runstadler Lab | Train dataset |
| A/environment/New Jersey/UGAI16-1048/2016(H16N3) | Dr. Rebecca Poulson | Train dataset |
| A/American green-winged teal/Minto Flats Alaska/19TL00405/2019(H1N1) | Runstadler Lab | Train dataset |
| A/Uruguay/716/2007(H3N2) | BEI Resources, NIAID, NIH | Train dataset |
| A/duck/Jiangsu/k1203/2010(H5N8) | SinoBiological | Train dataset |
| A/American green-winged teal/California/HKWF609/2007(H5N2) | SinoBiological | Train dataset |
| A/common teal/Netherlands/10/2000(H1N1) | BEI Resources, NIAID, NIH | Train dataset |
| A/mallard/Sweden/50/2002(H3N8) | BEI Resources, NIAID, NIH | Train dataset |
| A/mallard/Sweden/81/2002(H6N1) | BEI Resources, NIAID, NIH | Train dataset |
| A/mallard/Netherlands/1/1999(H4N6) | BEI Resources, NIAID, NIH | Train dataset |
| A/mallard/Sweden/24/2002(H8N4) | BEI Resources, NIAID, NIH | Train dataset |
| A/black headed gull/Sweden/1/1999(H13N6) | BEI Resources, NIAID, NIH | Train dataset |
| A/turkey/Italy/4602/1999(H7N1) | SinoBiological | Train dataset |
| A/chicken/VietNam/NCVD-016/2008(H5N1) | SinoBiological | Train dataset |
| A/chicken/Italy/22A/1998(H5N9) | SinoBiological | Train dataset |
| A/barnswallow/Hong Kong/D10-1161/2010(H5N1) | SinoBiological | Train dataset |
| A/Egypt/2321-NAMRU3/2007(H5N1) | SinoBiological | Train dataset |
| A/Yunnan/0127/2015(H5N6) | SinoBiological | Train dataset |
| A/Netherlands/219/2003(H7N7) | SinoBiological | Train dataset |
| A/American green-winged teal/Mississippi/300/2010(H11N9) | BEI Resources, NIAID, NIH | Train dataset |
| A/laughing gull/Delaware Bay/2718/1987(H9N5) | BEI Resources, NIAID, NIH | Train dataset |
| A/northern pintail/Interior Alaska/6MP0792/2006(H2N3) | Runstadler Lab | Train dataset |
| A/mallard/Interior Alaska/8MP0689/2008(H8N4) | Runstadler Lab | Train dataset |
| A/glaucous-winged gull/Southcentral Alaska/11JR02474/2011(H13N6) | Runstadler Lab | Train dataset |
| A/mallard/Southcentral Alaska/12ML01615/2014(H2N1) | Runstadler Lab | Train dataset |
| A/blue-winged teal/Missouri/15OS5111/2015(H2N9) | Runstadler Lab | Train dataset |
| A/mallard/Ohio/16OS0672/2016(H8N4) | Runstadler Lab | Train dataset |
| A/common goldeneye/Wisconsin/17OS5750/2017(H2N3) | Runstadler Lab | Train dataset |
| A/herring gull/Delaware/703/1988(H2N8) | SinoBiological | Train dataset |
| A/Canada/720/2005(H2N2) | SinoBiological | Train dataset |
| A/Japan/305/1957(H2N2) | SinoBiological | Train dataset |
| A/Anhui/1/2013(H7N9) | SinoBiological | Train dataset |
| A/pintail duck/Alberta/114/1979(H8N4) | SinoBiological | Train dataset |
| A/Hong Kong/35820/2009(H9N2) | SinoBiological | Train dataset |
| A/Hong Kong/3239/2008(H9N2) | SinoBiological | Train dataset |
| A/duck/Yangzhou/906/2002(H11N2) | SinoBiological | Train dataset |
| A/environment/Jiangxi/28/2009(H11N9) | SinoBiological | Train dataset |
| A/bar headed goose/Mongolia/143/2005(H12N3) | SinoBiological | Train dataset |
| A/black-headed gull/Netherlands/1/2000(H13N8) | SinoBiological | Train dataset |
| A/Australian shelduck/Western Australia/1756/1983(H15N2) | SinoBiological | Train dataset |
| A/China/CSKFQ-22-5/2022(H3N8) | GenScript | Train dataset |
| A/Fujiansiming/19/2021(H9N2) | GenScript | Train dataset |
| A/China/0428HA/2021(H10N3) | GenScript | Train dataset |
| A/sandpiper/Southcentral Alaska/16MB01220/2016(H5N2) | Runstadler Lab | Train dataset |
| A/sandpiper/Southcentral Alaska/16MB01156/2016(H5N2) | Runstadler Lab | Train dataset |
| A/duck/New_Zealand/76/1984(H9N1) | SinoBiological | Train dataset |
| Mutagenized H13 (ID B3) | Runstadler Lab | Train dataset |
| Mutagenized H13 (ID C1) | Runstadler Lab | Train dataset |
| Mutagenized H9 (ID E8) | Runstadler Lab | Train dataset |
| Mutagenized H14 (ID F5) | Runstadler Lab | Train dataset |

**Table S2. Glycan pairings from CFG microarrays.** Glycans are written using standard linear nomenclature (see National Center for Biotechnology Information for details^1^). Sp# indicates the spacer used to immobilize the glycan (see Grant *et al.* 2014 for details^2^).

| **Terminal Sialic Acid Conformation** | |
| --- | --- |
| **α2,3-linked** | **α2,6-linked** |
| Neu5Acα2-3GalNAcα-Sp8 | Neu5Acα2-6GalNAcα-Sp8 |
| Neu5Acα2-3GalNAcβ1-4GlcNAcβ-Sp0 | Neu5Acα2-6GalNAcβ1-4GlcNAcβ-Sp0 |
| Neu5Acα2-3Galβ-Sp8 | Neu5Acα2-6Galβ-Sp8 |
| Neu5Acα2-3Galβ1-4(6S)GlcNAcβ-Sp8 | Neu5Acα2-6Galβ1-4(6S)GlcNAcβ-Sp8 |
| Neu5Acα2-3Galβ1-4GlcNAcβ-Sp0 | Neu5Acα2-6Galβ1-4GlcNAcβ-Sp0 |
| Neu5Acα2-3Galβ1-4GlcNAcβ-Sp8 | Neu5Acα2-6Galβ1-4GlcNAcβ-Sp8 |
| Neu5Acα2-3Galβ1-4GlcNAcβ1-21-6(Neu5Acα2-3Galβ1-4GlcNAcβ1-21-3)Manβ1-4GlcNAcβ1-4GlcNAcβ-Sp12 | Neu5Acα2-6Galβ1-4GlcNAcβ1-2Manα1-6(Neu5Acα2-6Galβ1-4GlcNAcβ1-21-3)Manβ1-4GlcNAcβ1-4GlcNAcβ-Sp12 |
| Neu5Acα2-3Galβ1-4GlcNAcβ1-2Manα1-6(GlcNAcβ1-4)(Neu5Acα2-3Galβ1-4GlcNAcβ1-2Manα1-3)Manβ1-4GlcNAcβ1-4GlcNAcβ-Sp21 | Neu5Acα2-6Galβ1-4GlcNAcβ1-2Manα1-6(GlcNAcβ1-4)(Neu5Acα2-6Galβ1-4GlcNAcβ1-2Manα1-3)Manβ1-4GlcNAcβ1-4GlcNAcβ-Sp21 |
| Neu5Acα2-3Galβ1-4GlcNAcβ1-2Manα1-6(Neu5Acα2-3Galβ1-4GlcNAcβ1-2Manα1-3)Manβ1-4GlcNAcβ1-4(Fucα1-6)GlcNAcβ-6AA | Neu5Acα2-6Galβ1-4GlcNAcβ1-2Manα1-6(Neu5Acα2-6Galβ1-4GlcNAcβ1-2Manα1-3)Manβ1-4GlcNAcβ1-4(Fucα1-6)GlcNAcβ-6AA |
| Neu5Acα2-3Galβ1-4GlcNAcβ1-2Manα1-6(Neu5Acα2-3Galβ1-4GlcNAcβ1-2Manα1-3)Manβ1-4GlcNAcβ1-4(Fucα1-6)GlcNAcβ-Sp24 | Neu5Acα2-6Galβ1-4GlcNAcβ1-2Manα1-6(Neu5Acα2-6Galβ1-4GlcNAcβ1-2Manα1-3)Manβ1-4GlcNAcβ1-4(Fucα1-6)GlcNAcβ-Sp24 |
| Neu5Acα2-3Galβ1-4GlcNAcβ1-3GalNAc-Sp14 | Neu5Acα2-6Galβ1-4GlcNAcβ1-3GalNAc-Sp14 |
| Neu5Acα2-3Galβ1-4GlcNAcβ1-3Galβ1-3GlcNAcβ-Sp0 | Neu5Acα2-6Galβ1-4GlcNAcβ1-3Galβ1-3GlcNAcβ-Sp0 |
| Neu5Acα2-3Galβ1-4GlcNAcβ1-3Galβ1-4GlcNAcβ-Sp0 | Neu5Acα2-6Galβ1-4GlcNAcβ1-3Galβ1-4GlcNAcβ-Sp0 |
| Neu5Acα2-3Galβ1-4GlcNAcβ1-3Galβ1-4GlcNAcβ1-2Manα1-6(Neu5Acα2-3Galβ1-4GlcNAcβ1-3Galβ1-4GlcNAcβ1-2Manα1-3)Manβ1-4GlcNAcβ1-4GlcNAcβ-Sp12 | Neu5Acα2-6Galβ1-4GlcNAcβ1-3Galβ1-4GlcNAcβ1-2Manα1-6(Neu5Acα2-6Galβ1-4GlcNAcβ1-3Galβ1-4GlcNAcβ1-2Manα1-3)Manβ1-4GlcNAcβ1-4GlcNAcβ-Sp12 |
| Neu5Acα2-3Galβ1-4GlcNAcβ1-3Galβ1-4GlcNAcβ1-3GlcNAcα-Sp14 | Neu5Acα2-6Galβ1-4GlcNAcβ1-3Galβ1-4GlcNAcβ1-3GlcNAcα-Sp14 |
| Neu5Acα2-3Galβ1-4GlcNAcβ1-3Galβ1-4GlcNAcβ1-3Galβ1-4GlcNAcβ-Sp0 | Neu5Acα2-6Galβ1-4GlcNAcβ1-3Galβ1-4GlcNAcβ1-3Galβ1-4GlcNAcβ-Sp0 |
| Neu5Acα2-3Galβ1-4GlcNAcβ1-3Galβ1-4GlcNAcβ1-3Galβ1-4GlcNAcβ1-2Manα1-6(Neu5Acα2-3Galβ1-4GlcNAcβ1-3Galβ1-4GlcNAcβ1-3Galβ1-4GlcNAcβ1-2Manα1-3)Manβ1-4GlcNAcβ1-4GlcNAcβ-Sp12 | Neu5Acα2-6Galβ1-4GlcNAcβ1-3Galβ1-4GlcNAcβ1-3Galβ1-4GlcNAcβ1-2Manα1-6(Neu5Acα2-6Galβ1-4GlcNAcβ1-3Galβ1-4GlcNAcβ1-3Galβ1-4GlcNAcβ1-2Manα1-3)Manβ1-4GlcNAcβ1-4GlcNAcβ-Sp12 |
| Neu5Acα2-3Galβ1-4GlcNAcβ1-3Galβ1-4GlcNAcβ1-6(Galβ1-3)GlcNAcα-Sp14 | Neu5Acα2-6Galβ1-4GlcNAcβ1-3Galβ1-4GlcNAcβ1-6(Galβ1-3)GlcNAcα-Sp14 |
| Neu5Acα2-3Galβ1-4GlcNAcβ1-3Galβ1-4GlcNAcβ1-6(Neu5Acα2-3Galβ1-4GlcNAcβ1-3Galβ1-4GlcNAcβ1-3)GlcNAcα-Sp14 | Neu5Acα2-6Galβ1-4GlcNAcβ1-3Galβ1-4GlcNAcβ1-6(Neu5Acα2-6Galβ1-4GlcNAcβ1-3Galβ1-4GlcNAcβ1-3)GlcNAcα-Sp14 |
| Neu5Acα2-3Galβ1-4GlcNAcβ1-4Manα1-6(GlcNAcβ1-4)(Neu5Acα2-3Galβ1-4GlcNAcβ1-4(Neu5Acα2-3Galβ1-4GlcNAcβ1-2)Manα1-3)Manβ1-4GlcNAcβ1-4GlcNAcβ-Sp21 | Neu5Acα2-6Galβ1-4GlcNAcβ1-4Manα1-6(GlcNAcβ1-4)(Neu5Acα2-6Galβ1-4GlcNAcβ1-4(Neu5Acα2-6Galβ1-4GlcNAcβ1-2)Manα1-3)Manβ1-4GlcNAcβ1-4GlcNAcβ-Sp21 |
| Neu5Acα2-3Galβ1-4GlcNAcβ1-6(Galβ1-3)GlcNAcα-Sp14 | Neu5Acα2-6Galβ1-4GlcNAcβ1-6(Galβ1-3)GlcNAcα-Sp14 |
| Neu5Acα2-3Galβ1-4GlcNAcβ1-6(Neu5Acα2-3Galβ1-4GlcNAcβ1-2)Manα1-6(GlcNAcβ1-4)(Neu5Acα2-3Galβ1-4GlcNAcβ1-2Manα1-3)Manβ1-4GlcNAcβ1-4GlcNAcβ-Sp21 | Neu5Acα2-6Galβ1-4GlcNAcβ1-6(Neu5Acα2-6Galβ1-4GlcNAcβ1-2)Manα1-6(GlcNAcβ1-4)(Neu5Acα2-6Galβ1-4GlcNAcβ1-2Manα1-3)Manβ1-4GlcNAcβ1-4GlcNAcβ-Sp21 |
| Neu5Acα2-3Galβ1-4GlcNAcβ1-6(Neu5Acα2-3Galβ1-4GlcNAcβ1-2)Manα1-6(GlcNAcβ1-4)(Neu5Acα2-3Galβ1-4GlcNAcβ1-4(Neu5Acα2-3Galβ1-4GlcNAcβ1-2)Manα1-3)Manβ1-4GlcNAcβ1-4GlcNAcβ-Sp21 | Neu5Acα2-6Galβ1-4GlcNAcβ1-6(Neu5Acα2-6Galβ1-4GlcNAcβ1-2)Manα1-6(GlcNAcβ1-4)(Neu5Acα2-6Galβ1-4GlcNAcβ1-4(Neu5Acα2-6Galβ1-4GlcNAcβ1-2)Manα1-3)Manβ1-4GlcNAcβ1-4GlcNAcβ-Sp21 |
| Neu5Acα2-3Galβ1-4GlcNAcβ1-6(Neu5Acα2-3Galβ1-4GlcNAcβ1-3)GlcNAcα-Sp14 | Neu5Acα2-6Galβ1-4GlcNAcβ1-6(Neu5Acα2-6Galβ1-4GlcNAcβ1-3)GlcNAcα-Sp14 |
| Neu5Acα2-3Galβ1-4GlcNAcβ1-6GlcNAcα-Sp14 | Neu5Acα2-6Galβ1-4GlcNAcβ1-6GlcNAcα-Sp14 |
| Neu5Acα2-3Galβ1-4Glcβ-Sp0 | Neu5Acα2-6Galβ1-4Glcβ-Sp0 |
| Neu5Acα2-3Galβ1-4Glcβ-Sp8 | Neu5Acα2-6Galβ1-4Glcβ-Sp8 |

**Table S3. Convolutional neural network parameters and hyperparameters.**

| **Layer Name** | **Output Size** | **Kernel Size** | **Filters** | **Activation Function** |
| --- | --- | --- | --- | --- |
| Conv1D_0 | 8045 x 1 | 3 | 128 | Relu |
| MaxPooling1D_0 | 4022 x 128 | 3 stride 2 | - | - |
| BatchNormalization_0 | 4022 x 128 | - | - | - |
| Conv1D_1 | 4016 x 128 | 7 | 128 | Relu |
| MaxPooling1D_1 | 573 x 128 | 7 stride 7 | - | - |
| BatchNormalization_1 | 573 x 128 | - | - | - |
| Conv1D_2 | 571 x 128 | 3 | 128 | Relu |
| MaxPooling1D_2 | 571 x 128 | 1 stride 1 | - | - |
| BatchNormalization_2 | 571 x 128 | - | - | - |
| Conv1D_3 | 569 x 32 | 3 | 32 | Relu |
| MaxPooling1D_3 | 569 x 32 | 1 stride 1 | - | - |
| BatchNormalization_3 | 569 x 32 | - | - | - |
| Conv1D_4 | 559 x 128 | 11 | 128 | Relu |
| MaxPooling1D_4 | 559 x 128 | 1 stride 1 | - | - |
| BatchNormalization_4 | 559 x 128 | - | - | - |
| Flatten | 71552 | - | - | - |
| Dense_0 | 128 | - | 128 | Relu |
| Dropout = 0.5 | 128 | - | - | - |
| Dense_1 | 1 | - | 1 | Sigmoid |
| Conv1D = 1-dimensional convolutional layer | | | | |

**Table S4. Primers for amplification of hemagglutinin for assembly into a pcDNA3.1(+) vector.** All sequences are written 5’ to 3’. Sequence that anneals to the vector is italicized, NheI and BamHI restriction sites are underlined, and sequence that anneals to the HA insert is bolded.

| **Amplification target** | **Forward** | **Reverse** |
| --- | --- | --- |
| H9 | *TATAGGGAGACCCAAGCTGGCTAGC*TGGG**AGCAAAAGCAGGGGAAT** | *TCCACCACACTGGACTAGTGGATCC*TATT**AGTAGAAACAAGGGTGTTTT** |
| H16 | *TATAGGGAGACCCAAGCTGGCTAGC*TGGG**AGCAAAAGCAGGGGATA** | *TCCACCACACTGGACTAGTGGATCC*TATT**AGTAGAAACAAGGGTGTTTT** |
| Outside vector insertion site | *ACCCACTGCTTACTGGCTTA* | *TAGAAGGCACAGTCGAGGCT* |

**Table S5. Primers used for site directed mutagenesis of hemagglutinin.** Primers were designed using NEBaseChanger with parsimony. The base changes for each primer set are in bold.

| **Site Directed Mutagenesis Target** | **Forward** | **Reverse** | **Annealing Temp (°C)** |
| --- | --- | --- | --- |
| H9 G145S | AGCATGTTCA**AGT**TCATTCTACAG | CTGCTTGTTCCAGTGTAAG | 62 |
| H9 L226Q | TGTCAATGGT**CAG**CAGGGAAGAATTGATTATTATTG | AGGGGCCTTGGCCCT | 69 |
| H9 Q227G | CAATGGTCTG**GGG**GGAAGAATTGATTATTATTGG | ACAAGGGGCCTTGGC | 63 |
| H16 G159S | AAAACAGGAC**AGC**AAATATCCTGTTGTAAAAG | ACCAGCCAGACCAGA | 61 |
| H16 K160A | ACAGGACGG**GGC**ATATCCTGTTG | TTTACCAGCCAGACC | 59 |
| H16 G222K | GACCAGAATA**AAA**GACGGACAAAGG | CCAATCTCAAGTTCATAGC | 56 |
| H16 R227A | TGACGGACAA**GCG**AGTTGGATGAAAC | CCTATTCTGGTCCCAATC | 60 |
| H16 S228G | CGGACAAAGG**GGT**TGGATGAAAC | TCACCTATTCTGGTCCC | 62 |
| H16 K160A + R227A + G222K | GACCAGAATA**AAA**GACGGACAAGC | same as H16 G222K | 56 |
| H16 K160A + R227A + G222K + G159S | AAAACAGGAC**AGC**GCATATCCTGTTG | same as H16 G159S | 59 |
| H16 K160A + R227A + G222K + G159S + S228G | CGGACAAGCG**GGT**TGGATGAAAC | TCTTTTATTCTGGTCCCAATCTCAAG | 66 |

**References**

1. National Center for Biotechnology Information. Symbol Nomenclature for Glycans. 2024. <https://www.ncbi.nlm.nih.gov/glycans/snfg.html>

2. Grant OC, Smith HM, Firsova D, Fadda E, Woods RJ. Presentation, presentation, presentation! Molecular-level insight into linker effects on glycan array screening data. *Glycobiology*. 2014;24(1):17-25.
